# Neurogenic pulmonary edema in subarachnoid hemorrhage: relevant clinical concepts

**DOI:** 10.1186/s41984-021-00124-y

**Published:** 2021-11-15

**Authors:** Ivan David Lozada-Martínez, María Manuela Rodríguez-Gutiérrez, Jenny Ospina-Rios, Michael Gregorio Ortega-Sierra, Mauro Antonio González-Herazo, Lina Marcela Ortiz-Roncallo, Rafael Martínez-Imbett, Andrés Elías Llamas-Nieves, Tariq Janjua, Luis Rafael Moscote-Salazar

**Affiliations:** 1 Medical and Surgical Research Center, School of Medicine, University of Cartagena, Cartagena, Colombia; 2 Colombian Clinical Research Group in Neurocritical Care, School of Medicine, University of Cartagena, Cartagena, Colombia; 3 Latin American Council of Neurocritical Care, Cartagena, Colombia; 4 Global Committee Neurosurgery, World Federation of Neurosurgical Societies, Cartagena, Colombia; 5 Department of Medicine, Fundación Universitaria Visión de Las Americas, Pereira, Colombia; 6 Medical and Surgical Research Center, School of Medicine, Corporación Universitaria Rafael Nuñez, Cartagena, Colombia; 7 School of Medicine, Universidad de Cartagena, Cartagena, Colombia; 8 School of Medicine, Universidad de La Sabana, Bogotá, Colombia; 9 School of Medicine, Universidad del Norte, Barranquilla, Colombia; 10 Department of Intensive Care, Regions Hospital, Saint Paul, MN USA

**Keywords:** Subarachnoid hemorrhage, Pulmonary edema, Neurogenic inflammation, Lung injury, Treatments, Diagnosis

## Abstract

**Background:**

Subarachnoid hemorrhage (SAH) continues to be a condition that carries high rates of morbidity, mortality, and disability around the world. One of its complications is neurogenic pulmonary edema (NPE), which is mainly caused by sympathetic hyperactivity. Due to the complexity of the pathophysiological process and the unspecificity of the clinical presentation, it is little known by general practitioners, medical students and other health care workers not directly related to the neurological part, making the management of this chaotic condition difficult. This review aims to present recent evidence on clinical concepts relevant to the identification and management of NPE secondary to SAH.

**Main body of the abstract:**

NPE is defined as a syndrome of acute onset following significant central nervous system (CNS) injury. Its etiology has been proposed to stem from the release of catecholamines that produce cardiopulmonary dysfunction, with this syndrome being associated with spinal cord injury, cerebrovascular disorders, traumatic brain injury, status epilepticus, and meningitis. NPE has long been considered a rare event; but it may occur more frequently, mainly in patients with SAH. There are two clinical presentations of NPE: the early form develops in the first hours/minutes after injury, while the late form presents 12–24 h after neurological injury. Clinical manifestations consist of non-specific signs of respiratory distress: dyspnea, tachypnea, hypoxia, pink expectoration, crackles on auscultation, which usually resolve within 24–48 h in 50% of patients. Unfortunately, there are no tools to make the specific diagnosis, so the diagnosis is by exclusion. The therapeutic approach consists of two interventions: treatment of the underlying neurological injury to reduce intracranial pressure and control sympathetic hyperactivity related to the lung injury, and supportive treatment for pulmonary edema.

**Short conclusion:**

SAH is a severe condition that represents a risk to the life of the affected patient due to the possible complications that may develop. NPE is one of these complications, which due to the common manifestation of a respiratory syndrome, does not allow early and accurate diagnosis, being a diagnosis of exclusion. Therefore, in any case of CNS lesion with pulmonary involvement, NPE should be suspected immediately.

## Background

Subarachnoid hemorrhage (SAH) is a rare, severe neurological emergency that commonly affects patients with mean age older than 55 years [1]. This condition refers to bleeding into the subarachnoid space, which is located between the arachnoid and pia layers [2]. The most common cause that triggers this neurological disorder is trauma, and among the non-traumatic causes, 80% are generated by ruptured aneurysms of the intracranial circulation [3], although they can also occur secondary to other pathological entities such as Moya–Moya disease, arteriovenous malformations, vasculitis or amyloid angiopathy [3–5].

There is considerable variation in the annual incidence of SAH in different regions of the world. The global crude incidence of SAH was recently reported to be 7.9 per 100,000 persons per year, while in the United States the incidence was between 6 and 10 per 100,000 persons per year [6]. Among the large number of risk factors for the development of subarachnoid hemorrhage **(**Table 1), the most frequently associated sociodemographic factors are race (African Americans and Hispanics have a higher incidence than Caucasians), gender, and age (women between 40 and 60 years of age have a 1.2 higher risk of developing SAH than men) [6–11]. Up to 40% of patients with SAH will die and 50% to 66% will suffer permanent disability [12]. The prognosis of this event is evaluated according to the patient's age, pathological history, direct neurological involvement, and amount of bleeding at admission [13].

**Table 1:** Risk factors for the development of subarachnoid hemorrhage [6–11]

Non-modifiable	Modifiable
Alpha1-antitrypsin deficiency	Smoking (OR 3.1; 95% CI 2.7–3.5)
Sickle cell anemia	Arterial hypertension (OR 2.6; 95% CI 2.0–3.1)
First-degree relative with a history of SAH (OR 5.4; 95% CI 1.8–16.0)	Alcohol abuse (OR 1.5; 95% CI 1.3–1.8)
Polycystic kidney disease	Cocaine use
Female gender (HR 2.8; 95% CI 1.7–4.5)	Caffeine consumption

The modifiable risk factors associated with SAH that substantially increase the risk of this disease are hypertension, smoking, alcohol abuse and the use of sympathomimetics such as cocaine [7–11]. The risk of SAH is increased by the presence of an unruptured intracranial aneurysm (particularly those that are symptomatic, larger in size, and located in the posterior communicating artery or vertebrobasilar system), personal history of having suffered an SAH (with or without an untreated residual aneurysm), history of family aneurysms (at least 1 first-degree family member with an intracranial aneurysm and especially if ≥ 2 first-degree relatives are affected) [6], in addition genetic syndromes are also associated, such as autosomal dominant polycystic kidney disease and Ehlers-Danlos syndrome type IV [6–11].

Complications of SAH include hydrocephalus, vasospasm, epileptic seizures, intracranial hypertension, and extracranially, neurogenic pulmonary edema (NPE) [13, 14].

NPE is defined as a syndrome of acute onset following significant central nervous system (CNS) injury [15]. Its etiology has been proposed to stem from the release of catecholamines that produce cardiopulmonary dysfunction, with this syndrome being associated with spinal cord injury, cerebrovascular disorders, traumatic brain injury, status epilepticus, and meningitis [16]. NPE has long been considered a rare event; but it may occur more frequently, mainly in patients with SAH [17]. Despite being a serious complication that compromises the patient's life, it is poorly known and understood, with few epidemiological records existing to date [17]. The prevalence has been reported to be relatively low (2–5%) [18], however, accurate diagnosis of NPE is not always made, especially, due to the lack of etiology-specific diagnostic markers that may contribute to underdiagnosis, mismanagement, and lack of recording [19, 20]. Therefore, the true prevalence of NPE cannot be determined. In a study of patients with SAH due to aneurysmal rupture, the prevalence of clinically manifested pulmonary edema was 31% [16]. However, at autopsy, the true prevalence was found to be 78% [16].

Considering the lack of evidence on NPE and the impact of this complication on morbidity and mortality in patients with SAH, the aim of this manuscript is to present clinical and theoretical aspects for the understanding, diagnosis and management of this complication.

## Main text

### Pathophysiology

**Fig. 1:**
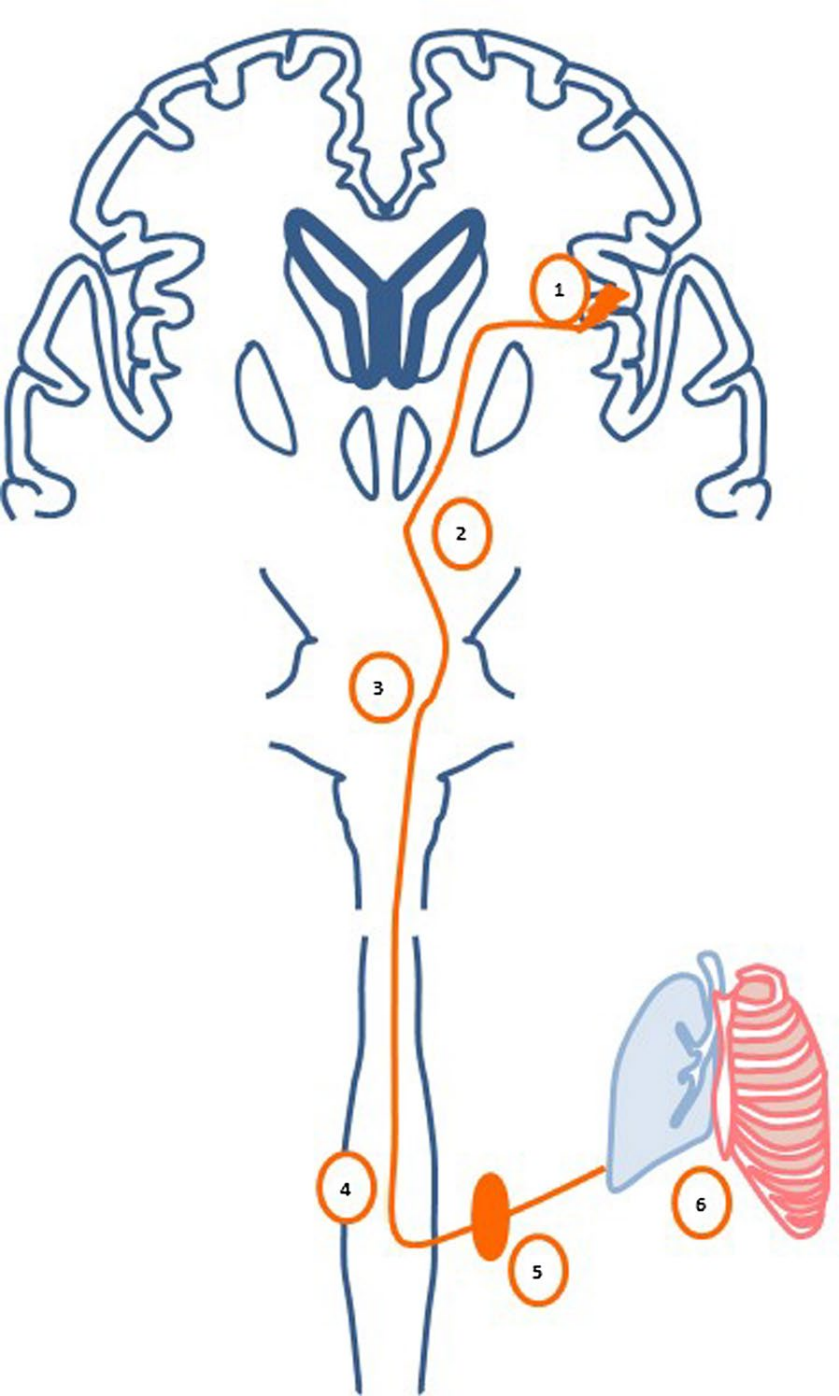
Involuntary control of breathing: Structures: 1. Anterior portion of the insular cortex; 2. Hypothalamus; 3. Stem centers: pneumotaxic center of the pons and ventral and dorsal respiratory groups of the pontobulbars (nucleus ambiguus and solitary nucleus, respectively), locus coeruleus; 4. Intermediolateral column of the spinal cord; 5. Superior cervical ganglion; 6. Bronchioalveolar receptors and accessory muscles of respiration. Control mechanism: The peripheral chemoreceptors of the carotid bulbs and aortic arch innervated by the glossopharyngeal nerve (activated by hypoxia, increased CO_2_, decreased arterial pH or hypoperfusion), project to the nucleus of the solitary tract, and this, together with the locus coeruleus, are central sensors of CO_2_ concentrations. These afferent nuclei plus the efferent nuclei in the ventral and dorsal pontobulbar groups have descending pathways to the intermediolateral column of the spinal cord, which contains sympathetic preganglionic neurons, in turn making efference through the superior cervical ganglion to the bronchi, alveoli, and accessory muscles of respiration. The structures of the indicated stem and the lateral intermediate column of the spinal cord receive superior control from the insula and hypothalamus, with sympathetic and parasympathetic autonomic control, which, when altered by central lesions, lead to the alteration of the predominantly sympathetic respiratory cycle. Created by authors

Although the pathophysiology of NPE is not well understood, among the most studied mechanisms are neurocardiac, neurohemodynamic, stadia theory and adrenergic hypersensitivity of pulmonary venules, which may occur simultaneously [19–24]. Following brain injury, hyperactivity of the sympathetic nervous system develops in the hypothalamus and medulla oblongata due to increased intracranial pressure, which is considered the main precipitating factor for the rest of the outcomes, by altering the nerve centers of respiration (Fig. 1) [19, 20]. The neuro-cardiac model associates’ sympathetic hyperactivity with increased circulation of catecholamines, which would produce a direct lesion of the cardiomyocyte, leading to the development of NPE. The neurohemodynamic mechanism postulates the indirect alteration of the myocardium by the sudden increase in systemic and pulmonary pressures following CNS lesion [19–21]. This hemodynamic state shifts blood from the systemic circulation (high resistance) to the pulmonary circulation where it is normally low resistance, thus increasing pulmonary blood volume and hydrostatic pressure and affecting pulmonary capillary permeability [19–21].

The burst theory states that the acute increase in capillary pressure injures the alveolar-capillary membrane, causing vascular leakage and protein-rich pulmonary edema. The mechanism of adrenergic hypersensitivity in pulmonary venules proposes a direct lesion on the endothelium of the pulmonary vascular network due to the presence of α- and β-receptors, which are affected by sympathetic hyperactivity [19–22].

Other determinants in the development of NPE include inflammatory mediators, cytokines released following brain injury that alter the permeability of pulmonary capillaries [23, 24]. Recent studies have described that the S100B protein produced at brain level (mainly by astrocytes), which physiologically intervenes in several processes such as protein phosphorylation, energy metabolism and calcium homeostasis, plays a fundamental role in the inflammatory process at extracerebral level [25–27]. In-vitro studies have shown that this protein stimulates the inflammatory response in different cell lineages through binding to the receptor for advanced glycation end products, which is widely expressed in alveolar type I cells, thus contributing to lung injury [25–27]. Although few studies describe the presence of clinical manifestations congruent with the hypotheses put forward for the pathophysiology of NPE, the biological plausibility between signaling pathways and inflammatory molecules and the pathological mechanisms that trigger lung injury is evident.

### Clinical manifestations

SAH classically presents with a burst headache, that is, one that reaches its greatest intensity in the first minute, occurring in up to 97% of people with this diagnosis [17, 27]. Depending on the time from headache onset to presentation, pain may resolve if the initial hemorrhage is small, called a sentinel hemorrhage [28]. These hemorrhages may occur 5–20 days before full presentation of SAH and are reported to be present in 10–40% of patients with aneurysmal SAH [29, 30]. It may also be accompanied by altered consciousness, nausea, vomiting, photophobia, seizures, meningismus or even death. Both seizures and loss of consciousness at the onset of hemorrhage have been found to be associated with a worse prognosis. Other less typical presenting signs may include acute encephalopathy and subdural hematoma [30–33].

There are two clinical presentations of NPE: the early form develops in the first hours/minutes after injury, while the late form presents 12–24 h after neurological injury [34–38]. Clinical manifestations consist of non-specific signs of respiratory distress: dyspnea, tachypnea, hypoxia, pink expectoration, crackles on auscultation, which usually resolve within 24–48 h in 50% of patients [37, 38]. In addition, the chest X-ray shows bilateral hyperdense infiltrate compatible with acute respiratory distress syndrome [37, 38]. However, observing that the clinical picture of this condition is nonspecific, the main thing is to suspect NPE in any patient with pulmonary involvement, in the presence or suspicion of CNS lesion.

### Diagnosis

Based on the nonspecific nature of the signs and symptoms at clinical presentation, the diagnosis of NPE is often made by exclusion. In the first instance, the existence of cardiogenic pulmonary edema in the context of neurological injury should be differentiated with echocardiographic help [39], or with the presence of the anamnesis that allow establishing cardiac, pulmonary or other history that may have an impact on the development of pulmonary edema [23, 25]. Other differential diagnoses to consider are aspiration pneumonia and ventilator-associated pneumonia in neurological patients with prolonged hospital stay [23–25]. Notwithstanding the above, general criteria can be used for the diagnosis of acute pulmonary edema, but in the context of CNS injury **(**Table 2**)** [40–42].

**Table 2:** Criteria for the diagnosis of neurogenic pulmonary edema [40–42]

Bilateral infiltrate
PaO_2_/FIO_2_ < 200
No evidence of left atrial hypertension
Presence of central nervous system lesion
Absence of other common causes of acute respiratory distress (aspiration, sepsis, blood transfusion, among others)
Clinical manifestations of pulmonary involvement

Studies suggest that patients with SAH develop electrocardiographic abnormalities, specifically Q wave or QS segment abnormalities and nonspecific T wave or ST segment changes, which could indicate the occurrence of NPE [43]. Another diagnostic tool is lung ultrasound, with a sensitivity of up to 90%, becoming an option for monitoring patients with SAH in intensive care units [44].

### Treatment

The therapeutic approach consists of two interventions: treatment of the underlying neurological injury to reduce intracranial pressure to control sympathetic hyperactivity related to the lung injury, and supportive treatment for pulmonary edema [23, 24, 27, 28, 45]. Within the supportive treatment of pulmonary impairment, monitoring of body fluids is difficult because maintaining adequate fluid volume is required for cerebral resuscitation, but the approach to neurogenic pulmonary edema requires fluid restriction [23, 24, 27, 28]. In this context, real-time lung ultrasound allows an assessment of respiratory failure, quantification and monitoring of pulmonary interstitial fluid, contributing to the management of liquid therapy [44]. Another viable intervention is the transpulmonary thermodilution technique [46], which consists of administering a saline solution at low temperature through a central venous catheter, then measuring the change in blood temperature and using this to construct a thermodilution curve that allows the calculation of hemodynamic parameters such as cardiac output and the pulmonary extravascular water index [46].

Supportive care involves adequate ventilation to provide sufficient oxygenation to prevent hypoxia and hypercapnia [27, 28, 45]. Therefore, the invasive or noninvasive ventilation technique must be individualized according to the severity of the patient's cardio-pulmonary and neurological affectation [23, 24]. Since patients present a life-threatening condition, it is important to be strict in the non-invasive monitoring of blood pressure, pulse oximetry, electrocardiography, echocardiography, radiography, among others [28, 45]. Extracorporeal membrane oxygenation can be used in patients with acute respiratory failure in whom mechanical ventilation and other therapies do not provide adequate gas exchange [47].

The usefulness of pharmacotherapy is varied, although no drug has been officially approved for routine use [23, 24, 28]. Hemodynamic instability with consequent organ hypoperfusion, metabolic acidosis, and progression of inflammation can be managed with vasoactive drugs [14–16]. Milrinone is used to increase cardiac output and is recommended when systolic blood pressure is > 90 mmHg. Dobutamine is preferred in those patients with blood pressure < 90 mmHg to increase cardiac output [14–16]. Other drugs that could be used according to the patient's context include diuretics such as furosemide, osmotic diuretics, and alpha-adrenergic blockers [48, 49].

### Future perspectives

In view of the large number of questions about the management, diagnosis and prevention of NPE, several hypotheses have been proposed [50–53]. Nastasovic et al. [50] conducted a study where they evaluated whether cardiac biomarkers had the ability to predict the development of NPE in patients with SAH, finding that out of 262 patients, 19 developed NPE, correlating the incidence of pulmonary edema with the severity of cerebrovascular disease [50]. Furthermore, in these patients there was greater myocardial injury (*p* < 0.000). Finally, they were able to demonstrate that elevated troponins (OR 4.980; 95% CI 1.27–19.49; *p* = 0.021) and leukocytosis (OR 22.195; 95% CI 3.99–123.50; *p* = 0.000), are predictors of NPE [50]. However, it is clear that the sample is insufficient to extrapolate these results, and the population was heterogeneous.

Regarding the prevention of NPE, it has been postulated that the administration of sevoflurane [51], caspase-1 inhibitor [52], and atropine [53] have the ability to prevent this complication. However, studies are insufficient and some have been performed only in non-human models [52]. Therefore, it is imperative to propose lines of research aimed at the in-depth description of the pathophysiological mechanism of this complication, its early diagnosis, prevention, treatment and prognosis, in order to modify decision-making algorithms and improve the survival rate of patients who suffer from SAH and develop extracranial complications.

## Conclusions

Subarachnoid hemorrhage is a severe condition that represents a risk to the life of the affected patient due to the possible complications that may develop. Neurogenic pulmonary edema is one of these complications, which due to the common manifestation of a respiratory syndrome, does not allow early and accurate diagnosis, being a diagnosis of exclusion. Therefore, in any case of central nervous system lesion with pulmonary involvement, neurogenic pulmonary edema should be suspected immediately. Treatment is mainly directed to the management of the triggering factor of subarachnoid hemorrhage, to stop the mechanisms that produce catecholaminergic discharge, in addition to providing ventilatory support with oxygen therapy.

## Data Availability

All data used are available from the corresponding author on request.

## Abbreviations

CNS: Central nervous system
NPE: Neurogenic pulmonary edema
SAH: Subarachnoid hemorrhage
